# The association between serum growth differentiation factor 15 levels and lower extremity atherosclerotic disease is independent of body mass index in type 2 diabetes

**DOI:** 10.1186/s12933-020-01020-9

**Published:** 2020-03-28

**Authors:** Xingxing He, Jiaorong Su, Xiaojing Ma, Wei Lu, Wei Zhu, Yufei Wang, Yuqian Bao, Jian Zhou

**Affiliations:** Department of Endocrinology and Metabolism, Shanghai Jiao Tong University Affiliated Sixth People’s Hospital; Shanghai Clinical Center for Diabetes; Shanghai Diabetes Institute; Shanghai Key Laboratory of Diabetes Mellitus, Shanghai, 200233 China

**Keywords:** Type 2 diabetes mellitus, Growth differentiation factor 15, Lower extremity atherosclerotic disease, Femoral intima-media thickness, Body mass index

## Abstract

**Background:**

Clinical and basic investigations have indicated a significant association between circulating growth differentiation factor 15 (GDF15) and cardiovascular disease; however, the relationship between GDF15 and lower extremity atherosclerotic disease (LEAD) has been less studied. The present study aimed to explore the association between GDF15 and LEAD in Chinese patients with type 2 diabetes mellitus (T2DM). Considering that obesity is an important factor associated with circulating GDF15 levels, whether the relationship between serum GDF15 levels and LEAD is affected by body mass index (BMI) was also analysed.

**Methods:**

A total of 376 hospitalized T2DM patients were enrolled (161 with LEAD and 215 without LEAD). A sandwich enzyme-linked immunosorbent assay was used to detect the serum GDF15 levels. The femoral intima-media thickness (F-IMT) and LEAD were assessed by ultrasonography.

**Results:**

Patients with LEAD had significantly higher serum GDF15 levels than those without LEAD, regardless of whether their BMI was < 25 kg/m^2^ or ≥ 25 kg/m^2^ (both *P *< 0.05). Serum GDF15 levels were independently positively related to the F-IMT (standardized *β *= 0.162, *P *= 0.002). After adjusting for confounding factors, per 1-standard deviation (SD) increase in the serum GDF15 levels was significantly related to an approximately 1.4-fold increased risk of LEAD in the total population (*P *< 0.05). Regardless of whether the BMI was < 25 kg/m^2^ or ≥ 25 kg/m^2^, this association remained significant, with approximately 1.6- and 1.4-fold increased risks of LEAD, respectively (both *P *< 0.05).

**Conclusions:**

High serum GDF15 levels were significantly correlated with an increased risk of LEAD in T2DM patients, and this relationship was independent of BMI.

## Background

Growth differentiation factor 15 (GDF15), also known as macrophage inhibitory factor-1, is a member of the transforming growth factor-β (TGF-β) cytokine superfamily [1]. Studies have found that GDF15 is a stress-responsive cytokine that is only highly expressed in the placenta [2] and prostate [3] and rarely expressed in myocardial tissues under normal physiological conditions. However, the expression and secretion of GDF15 can be strongly induced in the case of cardiovascular injury, such as pressure overload, myocardial infarction, heart failure, and atherosclerosis [4]. In recent years, a series of clinical studies [5–10] have reported that high circulating GDF15 levels are an independent predictive factor for several cardiovascular diseases in patients with atherosclerosis and acute coronary syndrome, and that investigating the levels of GDF15 could improve risk prediction beyond traditional risk factors and biochemical indicators.

Lower extremity atherosclerotic disease (LEAD) is one of the major vascular complications of diabetes and is associated with a substantial increase in the risk of non-fatal (such as gangrene, amputation, etc.) and fatal cardio- and cerebrovascular events [11, 12]. Furthermore, diabetes was observed to be independently associated with an increased risk of LEAD, depending on the severity of cardiovascular diseases [13]. Nevertheless, most previous related studies have focused on circulating GDF15 levels and myocardial or coronary artery diseases in patients with diabetes [14–16], and the relationship between GDF15 and LEAD has been less explored.

Therefore, in the present study, we hypothesized that serum GDF15 levels were correlated with LEAD as measured by ultrasonography in Chinese patients with type 2 diabetes mellitus (T2DM). Considering that obesity is an important factor associated with circulating GDF15 levels [17–19], we also analysed whether the relationship between serum GDF15 levels and LEAD was affected by body mass index (BMI).

## Materials and methods

### Study population

A total of 376 patients (259 men, 117 women) with T2DM who were hospitalized in the Department of Endocrinology and Metabolism of Shanghai Jiao Tong University Affiliated Sixth People’s Hospital were retrospectively included in this study during March 2015 and October 2019. The diagnosis of diabetes was based on the 2010 American Diabetes Association standards [20]. The inclusion criteria were patients with age from 20 to 80 years, 18.5 ≤ BMI < 30 kg/m^2^, and C-reactive protein levels < 10 mg/L. The exclusion criteria were patients who had severe cardiovascular diseases (*n *= 19), malignant tumours (*n *= 23), renal dysfunction (serum creatinine ≥ 115 µmol/L or glomerular filtration rate [GFR] < 60 mL/min/1.73 m^2^) (*n *= 49), and hepatic dysfunction (acute viral hepatitis, alanine aminotransferase, or aspartate aminotransferase > 1.5-fold the upper limit of normal) (*n *= 13). We collected the following clinical information from all the participants: diabetes duration, smoking status, alcohol consumption, and medication use. The study was approved by the Ethics Committee of Shanghai Jiao Tong University Affiliated Sixth People’s Hospital, and all participants provided written informed consent prior to enrolment.

### Anthropometric and biochemical assessments

BMI was calculated as weight/height^2^ (kg/m^2^). Systolic and diastolic blood pressure (SBP and DBP, respectively) were measured with a mercury sphygmomanometer after the subjects had rested for at least 10 min. Fasting venous blood samples were collected from subjects who had fasted overnight for 10 h, and 2-h venous blood samples were collected approximately 2 h after they ate breakfast. The levels of fasting plasma glucose (FPG), 2-h postprandial plasma glucose (2hPG), glycated haemoglobin A_1c_ (HbA_1c_), fasting C-peptide (FCP), 2-h C-peptide (2hCP), serum lipid profiles [including total cholesterol (TC), triglyceride (TG), high-density lipoprotein cholesterol (HDL-c), and low-density lipoprotein cholesterol (LDL-c)], haemoglobin (Hg) and C-reactive protein (CRP) were assessed with standard laboratory methods, as described previously [21]. The methods used to determine the GFR and homeostasis model assessment 2 of insulin resistance (HOMA2-IR) were also consistent with those used in a previous study [21].

Serum GDF15 levels were measured by a sandwich enzyme-linked immunosorbent assay (R & D Systems, Inc. Minneapolis, USA), with intra- and inter-assay coefficients of variation (CV) of 0.46–4.08% and 5.77–9.24%, respectively.

### Ultrasonography measurements and diagnosis of LEAD

Colour Doppler ultrasound examinations of the lower limb arteries were conducted using an Acuson Sequoia 512 scanner (Siemens Medical Solutions, Mountain View, CA, USA) equipped with a linear array transducer with frequencies of 5–13 MHz. The atherosclerotic plaque measurement range included the femoral artery, deep femoral artery, popliteal artery, superficial femoral artery, anterior tibial artery, posterior tibial artery and peroneal artery in each lower limb. According to the Mannheim Consensus [22], intima-media thickness (IMT) was assessed as the distance between the leading edge of the lumen-intima interface and the leading edge of the media-adventitia interface. An atherosclerotic plaque was defined as a focal structure encroaching into the arterial lumen at least 0.5 mm, 50% of the surrounding IMT value, or an IMT thickness ≥ 1.5 mm [22]. The mean value of the IMTs of the bilateral femoral arteries was defined as the femoral intima-media thickness (F-IMT). And the presence of atherosclerotic plaques in any of the lower limb artery segments listed above was defined as LEAD [23].

### Statistical analysis

SPSS version 22.0 was used for the statistical analysis. Data distributions were tested for normality. Variables with a normal distribution and F-IMT data are presented as the mean ± standard deviation (SD), while skewed data and categorical variables are expressed as the median (interquartile range: 25 to 75%) and percentages (%), respectively. An unpaired Student’s *t* test and Wilcoxon rank sum test were used to compare differences in normally distributed and skewed data, respectively. The Chi-square test was used for intergroup comparisons of categorical variables. Subgroup analysis was performed in populations with a BMI < 25 kg/m^2^ and a BMI ≥ 25 kg/m^2^, based on the 1998 World Health Organization criteria [24]. Multiple stepwise regression analysis was conducted to explore the factors influencing serum GDF15 levels. The values of serum GDF15 were standardized, and the relationship between the per 1-SD change in serum GDF15 levels and LEAD was analysed by logistic regression analysis. The level of statistical significance was set at a two-tailed *P* value less than 0.05.

## Results

### Clinical characteristics of the study participants

A total of 376 T2DM patients with a median age of 48 (38–58) years, including 259 men and 117 women, were included in this study. The study population was divided into four subgroups based on BMI 25 kg/m^2^ and the presence of LEAD. The clinical characteristics of the subjects are summarized in Table 1. Whether patients had a BMI < 25 kg/m^2^ or ≥ 25 kg/m^2^, subjects with LEAD exhibited older age, higher levels of F-IMT, longer diabetes duration, and a higher usage of lipid-lowering therapy but a lower GFR than those without LEAD (all *P *< 0.05, Table 1).

**Table 1:** Characteristics of the study participants

Variable	BMI < 25 kg/m^2^		BMI ≥ 25 kg/m^2^	
	With LEAD (*n* = 89)	Without LEAD (*n* = 95)	With LEAD (*n* = 72)	Without LEAD (*n* = 120)
*n* (men/women)	58/31	59/36	55/17	87/33
Age (years)	59 (53–65)**	42 (35–51)	58 (50–63)**	40 (33–46)
BMI (kg/m^2^)	23.51 (22.50–24.22)**	22.60 (21.23–23.88)	26.88 (25.74–28.25)	27.34 (26.21–28.27)
SBP (mmHg)	120 (115–131)	120 (112–130)	128 (120–140)	126 (120–138)
DBP (mmHg)	77 (70–82)	80 (70–85)	80 (76–90)	80 (76–90)
TC (mmol/L)	4.87 ± 1.28	4.73 ± 1.04	4.80 ± 1.18	4.95 ± 1.11
TG (mmol/L)	1.28 (0.97–1.98)	1.21 (0.78–1.97)	1.90 (1.23–2.64)	2.00 (1.38–2.74)
HDL-c (mmol/L)	1.12 (0.87–1.33)	1.08 (0.94–1.30)	0.93 (0.84–1.10)	0.96 (0.81–1.11)
LDL-c (mmol/L)	2.99 ± 1.14	2.92 ± 0.81	3.00 ± 0.95	3.03 ± 0.94
CRP (mg/L)	0.84 (0.45–1.74)	0.65 (0.30–1.42)	1.07 (0.50–1.88)	1.25 (0.69–2.21)
FPG (mmol/L)	7.69 (6.62–10.22)	7.50 (6.19–8.95)	9.11 (6.94–10.64)	7.95 (6.89–10.03)
2hPG (mmol/L)	12.74 (10.38–15.14)	12.38 (9.21–15.23)	13.67 (9.72–16.81)	13.31 (10.85–15.91)
HbA_1c_ (%)	10.0 (8.1–11.2)	9.3 (7.2–10.8)	9.8 (8.2–10.8)	9.5 (7.7–11.3)
FCP (ng/mL)	1.35 (1.03–1.94)	1.50 (0.82–2.04)	1.78 (1.31–2.26)	1.84 (1.29–2.64)
2hCP (ng/mL)	2.64 (1.74–3.78)	2.90 (1.70–4.57)	3.33 (2.39–4.48)	3.83 (2.32–6.50)
HOMA2-IR	1.18 (0.83–1.66)	1.29 (0.70–1.75)	1.53 (1.17–2.10)	1.60 (1.15–2.24)
Hg (g/L)	142 (132–150)*	145 (137–154)	148 (135–154)	148 (139–155)
GFR (mL/min/1.73 m^2^)	94.80 (83.00–110.70)**	104.50 (92.70–121.56)	93.79 (79.95–108.58)**	107.66 (89.18–124.33)
Diabetes duration (years)	10.00 (6.00–14.00)**	6.00 (2.00–10.00)	10.50 (5.00–14.00)**	4.00 (0.33–9.00)
F-IMT (mm)	0.85 ± 0.00**	0.60 ± 0.03	0.85 ± 0.01**	0.60 ± 0.03
Current smoking, *n* (%)	35 (39.33)	25 (26.32)	24 (33.33)	41 (34.17)
Current alcohol consumption, *n* (%)	14 (15.73)	20 (21.05)	14 (19.44)	30 (25.00)
Anti-hypertensive therapy, *n* (%)	39 (43.82)**	18 (18.95)	31 (43.06)	39 (32.50)
Lipid-lowering therapy, *n* (%)	71 (79.78)**	46 (48.42)	59 (81.94)**	73 (60.83)

Data are expressed as mean ± standard deviation, median (interquartile range: 25 to 75%), or *n* (%)

*BMI* body mass index, *SBP* systolic blood pressure, *DBP* diastolic blood pressure, *TC* total cholesterol, *TG* triglyceride, *HDL*-*c* high-density lipoprotein cholesterol, *LDL*-*c* low-density lipoprotein cholesterol, *CRP* C-reactive protein, *FPG* fasting plasma glucose, *2hPG* 2-h plasma glucose, *HbA*_*1c*_ glycated haemoglobin A_1c_, *FCP* fasting C-peptide, *2hCP* 2-h C-peptide, *HOMA2*-*IR* homeostasis model assessment 2 of insulin resistance, *Hg* haemoglobin, *GFR* glomerular filtration rate, *F*-*IMT* femoral intima-media thickness

* *P* < 0.05 versus without LEAD

** *P* < 0.01 versus without LEAD

The median (25th, 75th percentile) serum GDF15 levels among the entire study population was 796.78 (546.46–1224.60) pg/mL. No significant difference was found between men and women [792.11 (566.44–1254.75) pg/mL versus 814.98 (524.73–1196.70) pg/mL, *P *= 0.532]. Those with LEAD had significantly higher levels of GDF15 than those without LEAD [910.29 (604.11–1379.41) pg/mL versus 718.43 (528.75–1108.79) pg/mL, *P *= 0.002].

### Comparison of serum GDF15 levels between patients with a BMI < 25 kg/m^2^ and those with a BMI ≥ 25 kg/m^2^

**Fig. 1:**
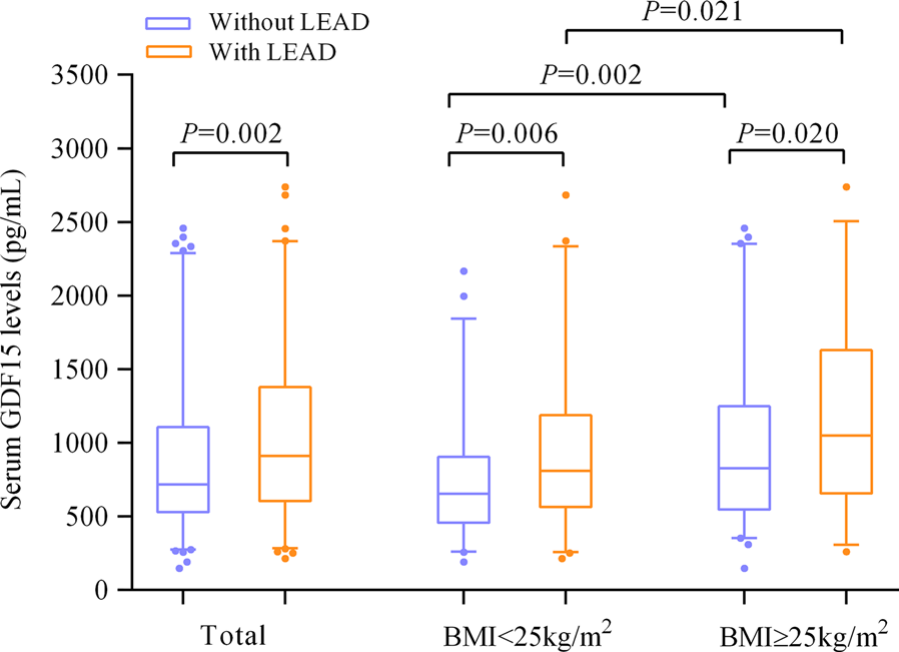
Comparison of serum GDF15 levels. Data are shown as the median with 2.5th and 97.5th percentiles

Compared with patients with a BMI < 25 kg/m^2^, patients with a BMI ≥ 25 kg/m^2^ exhibited much higher serum GDF15 levels [863.87 (587.51–1363.77) pg/mL versus 691.78 (521.30–1093.18) pg/mL, *P *= 0.001]. Regardless of LEAD status, subjects with a BMI ≥ 25 kg/m^2^ had significantly higher levels of GDF15 than subjects with a BMI < 25 kg/m^2^ (both *P *< 0.05, Fig. 1). Additionally, the serum GDF15 levels were observed to be significantly higher in patients with LEAD than in those without LEAD, regardless of whether their BMI was < 25 kg/m^2^ or ≥ 25 kg/m^2^ (both *P *< 0.05, Fig. 1).

### Correlations of serum GDF15 levels with F-IMT and other metabolic parameters

Spearman’s correlation analysis showed that the serum GDF15 levels were positively associated with F-IMT (*r *= 0.164, *P *= 0.001). In addition, the serum GDF15 levels were also positively correlated with age (*r *= 0.205, *P *< 0.001), BMI (*r *= 0.179, *P *< 0.001), FCP (*r *= 0.110, *P *= 0.033), HOMA2-IR (*r *= 0.103, *P *= 0.046), TG (*r *= 0.130, *P *= 0.012), and diabetes duration (*r *= 0.257, *P *< 0.001), but negatively correlated with TC (*r *= − 0.214, *P *< 0.001), LDL-c (*r *= − 0.206, *P *< 0.001), and HDL-c (*r *= − 0.142, *P *= 0.006).

Multivariate stepwise regression analysis defined the serum GDF15 levels as the dependent variable, and gender, BMI, SBP, DBP, HbA_1c_, HOMA2-IR, Hg, TC, TG, HDL-c, LDL-c, CRP, GFR, F-IMT, current smoking status, and current alcohol consumption status were designated as the independent variables. The results showed that F-IMT (standardized *β *= 0.162, *P *= 0.002) and BMI (standardized *β *= 0.193, *P *< 0.001) were independently and positively associated with serum GDF15 levels. In addition, LDL-c (standardized *β *= − 0.263, *P *< 0.001) was negatively associated with the serum GDF15 levels (Table 2).

**Table 2:** Multivariate regression analyse of factors associated with serum GDF15 levels

Variable	Standardized *β*	*t*	*P*
LDL-c	− 0.263	− 5.187	< 0.001
BMI	0.193	3.804	< 0.001
F-IMT	0.162	3.196	0.002

Independent variables originally included: gender, BMI, SBP, DBP, HbA_1c_, HOMA2-IR, Hg, TC, TG, HDL-c, LDL-c, CRP, GFR, F-IMT, current smoking, current alcohol consumption

*BMI* body mass index, *SBP* systolic blood pressure, *DBP* diastolic blood pressure, *HbA*_*1c*_ glycated hemoglobin A_1c_, *HOMA2*-*IR* homeostasis model assessment 2 of insulin resistance, *Hg* hemoglobin, *TC* total cholesterol, *TG* triglyceride, *HDL*-*c* high-density lipoprotein cholesterol, *LDL*-*c* low-density lipoprotein cholesterol, *CRP* C-reactive protein, *GFR* glomerular filtration rate, *F*-*IMT* femoral intima-media thickness, *GDF15* serum growth differentiation factor 15

### Relationships between serum levels of GDF15 (per 1-SD increase) and LEAD

**Fig. 2:**
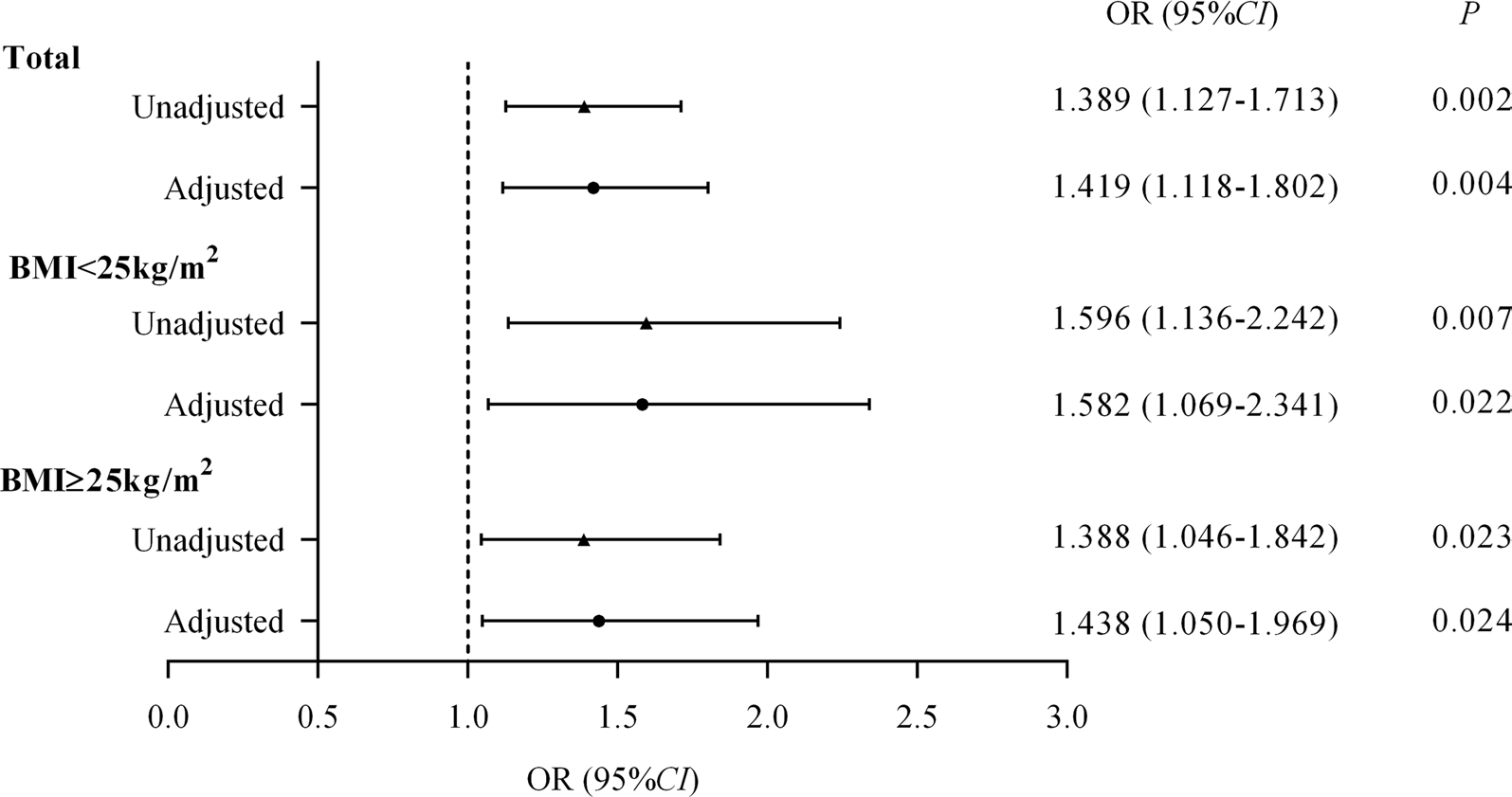
Relationships between serum levels of GDF15 (per 1-SD increase) and LEAD in the total population, patients with a BMI < 25 kg/m^2^, and patients with a BMI ≥ 25 kg/m^2^. Independent variables in the adjusted models included: gender, SBP, DBP, HbA_1c_, CRP, TC, TG, HDL-c, LDL-c, HOMA2-IR, GFR, GDF15, current smoking status, current alcohol consumption status, anti-hypertensive therapy, and lipid-lowering therapy

Defining LEAD as the dependent variable, the present study constructed three logistic regression models to further determine the relationship between serum GDF15 levels and LEAD in the total population, patients with a BMI < 25 kg/m^2^, and patients with a BMI ≥ 25 kg/m^2^. It was observed that per 1-SD increase in serum GDF15 levels was associated with an increased risk of LEAD in all three groups, with OR values of 1.389 (*95% CI* 1.127–1.713, *P *= 0.002), 1.596 (*95% CI* 1.136–2.242, *P *= 0.007), and 1.388 (*95% CI* 1.046–1.842, *P *= 0.023) in the total population, patients with a BMI < 25 kg/m^2^, and patients with a BMI ≥ 25 kg/m^2^, respectively. After multivariable adjustment (including gender, SBP, DBP, HbA_1c_, CRP, TC, TG, HDL-c, LDL-c, HOMA2-IR, GFR, current smoking status, current alcohol consumption status, anti-hypertensive therapy, and lipid-lowering therapy), per 1-SD increase in serum GDF15 levels remained significantly related to LEAD, with 1.419 (1.118–1.802)-fold increased odds in the total population, and 1.582 (1.069–2.341)- and 1.438 (1.050–1.969)-fold increased odds in patients with a BMI < 25 kg/m^2^ and patients with a BMI ≥ 25 kg/m^2^, respectively (all *P *< 0.05, Fig. 2).

In addition, the logistic regression models also showed that in all three groups, the GFR was independently negatively and lipid-lowering therapy was independently positively associated with LEAD (all *P *< 0.05).

## Discussion

The present study was the first to explore the relationship between serum GDF15 levels and LEAD in Chinese patients with T2DM. The results showed that patients with LEAD exhibited much higher serum GDF15 levels than those without LEAD, regardless of whether they had a BMI < 25 kg/m^2^ or ≥ 25 kg/m^2^. The F-IMT was identified as an independent factor positively associated with serum GDF15 levels. High serum GDF15 levels were significantly correlated with an increased risk of LEAD, and this relationship was independent of BMI.

LEAD is the manifestation of peripheral atherosclerotic disease and is characterized as the stenosis and occlusion of lower limb arteries [25]. Due to the similar pathophysiological mechanisms (such as oxidative stress and endothelial dysfunction) between LEAD and atherosclerosis in other vasculature, LEAD is closely related to the occurrence of cardiovascular disease [11, 12, 26]. In addition, diabetes-associated LEAD is often concomitant with neuropathy [27]. The decrease in the sense of pain and temperature caused by diabetic neuropathy may conceal the symptoms of LEAD (e.g., resting pain), leading to a delayed diagnosis of LEAD and more serious outcomes such as amputations [28, 29]. Considering the heavy public health and economic burden and the worse prognosis caused by diabetes-related amputations [30], early detection or exploration of an effective biomarker for LEAD is needed.

The results from previous studies have shown that increased circulating GDF15 levels were closely associated with the development and progression of various cardiovascular diseases (including myocardial ischaemia/reperfusion, heart failure, atherosclerosis, and acute coronary syndrome), and were shown to be a strong and independent predictor of mortality and disease progression in patients with atherosclerosis and coronary artery disease [5–10, 31–36]. In diabetic patients, higher levels of lipids and chronic inflammation may directly damage the cardiovascular system [37]. As expected, circulating GDF15 levels have also been shown to help identify and predict cardiovascular disease risk in patients with diabetes [16, 38, 39]. In a study with newly diagnosed T2DM patients, Shin et al. [16] used the Framingham risk score and the New Pooled Cohort Equation score to estimate the 10-year risk of atherosclerotic cardiovascular disease. After adjusting for traditional risk factors, GDF15 was significantly positively correlated with both the Framingham risk score and the New Pooled Cohort Equation score, indicating that the levels of GDF15 may be a useful predictive biomarker of cardiovascular risk in newly diagnosed T2DM patients. Another study conducted by Dominguez-Rodriguez et al. demonstrated that elevated GDF15 levels could be valuable as a unique independent predictor of diabetic cardiomyopathy. Additionally, the results from receiver operating characteristic curves indicated that GDF15 represents a useful and novel tool to screen for diabetic cardiomyopathy in patients with T2DM [39].

However, the correlation between GDF15 and diabetic cardiovascular complications has mostly been investigated with regard to myocardial or coronary artery diseases, and few data have been reported about GDF15 and LEAD. To the best of our knowledge, this is the first study to investigate an association between serum GDF15 levels and LEAD in T2DM patients. Given that obesity contributes substantially to cardiovascular and other health risks [40], and is one of the most important factors associated with circulating GDF15 levels [17–19], the present study used a BMI cut-off of 25 kg/m^2^ to assess normal weight or overweight/obesity and analysed the influence of BMI on the relationship between serum GDF15 levels and LEAD. Our results showed that serum GDF15 levels were positively correlated with LEAD, regardless of whether the BMI was < 25 kg/m^2^ or ≥ 25 kg/m^2^. After adjusting for confounding factors, per 1-SD increase in the serum GDF15 levels was observed to be significantly related to an approximately 1.4-fold increased risk of LEAD in the total population. Moreover, regardless of whether BMI was < 25 kg/m^2^ or ≥ 25 kg/m^2^, this association remained significant, with approximately 1.6- and 1.4-fold increased risks of LEAD, respectively. These results indicated that the association between serum GDF15 levels and LEAD is independent of BMI in T2DM patients.

The mechanism underlying the relationship between serum GDF15 levels and LEAD remains to be determined. On the one hand, elevated GDF15 has been shown to promote inflammation and angiogenesis, implying that GDF15 may play an important role in the pathogenesis of atherosclerosis [41, 42]. However, meanwhile, a reduction in early atherosclerotic lesion size was observed after 4 weeks on a Western-type diet in GDF15-deficient mice; however, no striking differences in lesion size were noticed at the 12-week time point [42]. Similar results were reported in an ApoE(−/−) mouse model of atherosclerosis [43], indicating that GDF15 may play an anti-inflammatory role in the process of atherosclerosis. The expression of GDF15 can be upregulated by multiple proinflammatory stimuli in macrophages, including tumour necrosis factor-α, interleukin (IL)-1b, and IL-2; however, GDF15, in turn, can inhibit the activation of macrophages [1]. Thus, GDF15 functions as a pro- and anti-inflammatory factor in the development and progression of atherosclerosis, and its activity may depend on the pathophysiological environment and progression stage [4].

The present study was limited by the retrospective nature and relatively small sample size, and the cause–effect relationship between the serum GDF15 levels and LEAD and the predictive value of the serum GDF15 levels for LEAD need to be confirmed in further larger prospective studies including nondiabetic populations. Additionally, the present study design did not detect the ankle-brachial index and symptoms of intermittent claudication or ischaemic resting pains, and age was not matched in patients with and without LEAD; these factors need to be addressed in future studies to further explore the association between GDF15 and LEAD. Lastly, the body composition is quite different in Asia/China than in the United States/Europe, so these results may not be applicable to non-Asian populations.

## Conclusions

In conclusion, among Chinese patients with T2DM, serum GDF15 levels were significantly elevated in those with LEAD. High serum GDF15 levels were significantly correlated with an increased risk of LEAD, and this relationship was independent of BMI. This suggests that GDF15 may be a potential biomarker to identify individuals with a higher risk of LEAD in clinical practice.

## Data Availability

The datasets used and analysed during the current study are not publicly available due to the individual privacy of the patients included in this study, but are available from the corresponding author on reasonable request.

## Abbreviations

BMI: Body mass index
CRP: C-reactive protein
DBP: Diastolic blood pressure
FCP: Fasting C-peptide
F-IMT: Femoral intima-media thickness
FPG: Fasting plasma glucose
GDF15: Growth differentiation factor 15
GFR: Glomerular filtration rate
HbA_1c_: Glycated haemoglobin A_1c_
HDL-c: High-density lipoprotein cholesterol
Hg: Haemoglobin
HOMA2-IR: Homeostasis model assessment 2 of insulin resistance
IMT: Intima-media thickness
LDL-c: Low-density lipoprotein cholesterol
LEAD: Lower extremity atherosclerotic disease
SBP: Systolic blood pressure
SD: Standard deviation
T2DM: Type 2 diabetes mellitus
TC: Total cholesterol
TG: Triglyceride
2hCP: 2-h C-peptide
2hPG: 2-h plasma glucose
